# Die COVID-19-Pandemie – Wie hat sie die Kinderpsyche beeinflusst?

**DOI:** 10.1007/s00112-023-01775-x

**Published:** 2023-06-06

**Authors:** Ulrike Ravens-Sieberer, Anne Kaman, Janine Devine, Franziska Reiß

**Affiliations:** Zentrum für Psychosoziale Medizin, Klinik für Kinder- und Jugendpsychiatrie, -psychotherapie und -psychosomatik, Forschungssektion Child Public Health, Universitätsklinikum Hamburg-Eppendorf, Martinistraße 52, 20246 Hamburg, Deutschland

**Keywords:** COPSY-Studie, Psychische Gesundheit, Gesundheitsbezogene Lebensqualität, Soziale Isolation, Längsschnittstudien, COPSY study, Mental health, Health-related quality of life, Social isolation, Longitudinal studies

## Abstract

**Hintergrund:**

Lockdowns, Kontaktbeschränkungen, Schließung von Kinderbetreuungs‑, Schul- und Freizeiteinrichtungen während der COVID-19-Pandemie haben den Alltag von Kindern und Jugendlichen deutlich beeinträchtigt.

**Ziel der Arbeit (Fragestellung):**

Der Beitrag untersucht die Auswirkungen der Pandemie auf die psychische Gesundheit von Kindern und Jugendlichen in Deutschland.

**Material und Methoden:**

Die bevölkerungsbezogene COPSY-Längsschnittstudie (*Co*rona und *Psy*che) umfasst bisher 5 Befragungszeitpunkte (t1: Mai bis Juni 2020 bis t5: Sept. bis Okt. 2022). Es wurden etwa 1600 Eltern von 7‑ bis 17-Jährigen sowie 1000 Kinder und Jugendliche zwischen 11 und 17 Jahren mithilfe etablierter Instrumente zur psychischen Gesundheit befragt. Präpandemische Vergleichsdaten lieferte die repräsentative BELLA-Studie (Befragung zum seelischen Wohlbefinden und Verhalten).

**Ergebnisse:**

Während der Pandemie stieg der Anteil von Kindern und Jugendlichen, die über eine geminderte gesundheitsbezogene Lebensqualität berichteten, von 15 % (präpandemisch) auf 48 % im ersten Jahr an und reduzierte sich 2022 auf 27 %. Damit war der Wert noch deutlich über dem vor Beginn der Pandemie. Ein ähnlicher Verlauf zeigte sich für psychische Auffälligkeiten: Deren Prävalenz stieg von 18 % (präpandemisch) auf 31 % an und reduzierte sich 2022 auf 23 %. Symptome für Ängstlichkeit und Depressivität folgten dem Trend. Lediglich Symptome der Depressivität sanken auf das präpandemische Niveau zurück. Hinsichtlich ihres Gesundheitsverhaltens bewegt sich ein Drittel der Kinder und Jugendlichen noch immer zu wenig.

**Diskussion:**

Die psychische Gesundheit von Kindern und Jugendlichen wurde während der Pandemie lange ignoriert. Dringend müssen Unterstützung und finanzielle Mittel gewährt werden, um negative psychische Gesundheitseffekte zu reduzieren und Beeinträchtigungen durch erneute Krisen vorzubeugen.

## Abstract (en)

**Background:**

The COVID-19 pandemic has had a substantial impact on the daily life of children and adolescents due to lockdowns, contact restrictions, closings of childcare, schools and leisure facilities.

**Objective:**

This article examines the impact of the pandemic on the mental health of children and adolescents in Germany.

**Material and methods:**

In the population-based longitudinal COPSY study (COVID-19 and Psychological Health) 1600 parents of children aged 7–17 years old and 1000 children and adolescents aged 11–17 years old completed established questionnaires at 5 online survey points (t1: May and June 2020 to t5: September and October 2022). For comparison, pre-pandemic data were obtained from the representative BELLA study.

**Results:**

During the pandemic the portion of children and adolescents, who reported a low health-related quality of life, increased from pre-pandemic 15% to 48% in the first year of the pandemic and improved in year 3 to 27%. This is still substantially above pre-pandemic levels. Similar trajectories were found for mental health problems which rose from 18% (pre-pandemic) to 31% and improved in year 3 to 23%. For anxiety and depression, the changes were similar except that depressive symptoms returned to pre-pandemic levels in year 3. The physical activity of almost one third of the youth was still very low in year 3.

**Conclusion:**

The mental health of children and adolescents was ignored during the pandemic for a long time. Now support and allocation of funding is urgently needed to mitigate the negative mental health effects and prevent further impairment by new crises.

Mit Beginn der COVID-19-Pandemie Anfang 2020 erlebten die Menschen weltweit erhebliche Herausforderungen und Einschränkungen im Alltag. Kinder und Jugendliche waren aufgrund ihrer entwicklungsbedingten Vulnerabilität von den Auswirkungen der Pandemie besonders betroffen. Neuerdings belasten auch der Ukraine-Krieg, die Inflation, die Klima- und die Energiekrise die Kinderseelen. Evidenzbasierte Daten zur psychischen Gesundheit von Kindern und Jugendlichen in Krisenzeiten werden dringend benötigt, um wissenschaftlich fundiert gesellschaftspolitische und gesundheitsbezogene Entscheidungen zu treffen.

## Hintergrund

Aus internationalen Reviews und Metaanalysen ist bekannt, dass seelische Belastungen und psychische Auffälligkeiten bei Kindern und Jugendlichen während der COVID-19-Pandemie zugenommen haben. Vor allem im ersten Jahr der Pandemie hat sich die psychische Gesundheit von Kindern und Jugendlichen internationalen Übersichtsarbeiten zufolge erheblich verschlechtert [7, 15, 22].

Die Mehrzahl der Studien zur psychischen Gesundheit von Kindern in Deutschland wurde im ersten Pandemiejahr (2020) durchgeführt. Das Belastungserleben variierte abhängig von den Pandemiewellen und deren Beschränkungen [23]. Zu Beginn der Pandemie berichten mehrere Studien über eine deutliche Abnahme der Lebensqualität von Kindern und Jugendlichen hierzulande [1, 16, 23, 28], die über den Winter 2020 [19] bis zum Frühjahr 2021 [27] anhielt. Erst im Herbst 2021 verbesserte sich die Lebensqualität wieder [16].

Um Aussagen über die Entwicklung und Langzeitfolgen treffen zu können, werden prospektive Kohortenstudien benötigt. Sie erfassen die psychische Gesundheit von Kindern und Jugendlichen längsschnittlich und multiperspektivisch nach international vergleichbaren Kriterien.

Für Deutschland untersucht die COPSY-Längsschnittstudie (*Co*rona und *Psy*che) seit Mai 2020 die Auswirkungen der Pandemie auf die psychische Gesundheit und das Wohlbefinden von Kindern und Jugendlichen. An bisher 5 Zeitpunkten (t1: Mai bis Juni 2020, t2: Dezember 2020 bis Januar 2021, t3: September bis Oktober 2021, t4: Februar 2022, t5: September bis Oktober 2022) wurde erfragt, wie es den Kindern und Jugendlichen ging. Für jede Erhebung wurden 1600 bis 1700 Eltern von 7‑ bis 17-Jährigen und 1000 Kinder und Jugendliche im Alter von 11 bis 17 Jahren online befragt (50–52 % Mädchen, Eltern: 44 bis 46 Jahre alt, 83 % ohne Migrationshintergrund, 57 % mittlerer Bildungsabschluss, 52–53 % in Vollzeit tätig, 29–31 % in Teilzeit. Es hatten 60–62 % der Kinder und Jugendlichen zu dem Zeitpunkt bereits eine Infektion mit dem Severe Acute Respiratory Syndrome Coronavirus 2 [SARS-CoV-2] durchgemacht).

Die COPSY-Studie ist eine kombinierte Quer- und Längsschnittstudie: Für jede Befragung wurden neue Teilnehmenden gewonnen (repräsentativer Querschnitt) sowie Teilnehmende aus den vorherigen Befragungszeitpunkten erfasst (Längsschnitt). In allen Befragungen wurden standardisierte Fragebögen zur psychischen Gesundheit, zum Wohlbefinden und zum Gesundheitsverhalten von Kindern und Jugendlichen eingesetzt [20]. Die Ergebnisse der 5 Befragungszeitpunkte der COPSY-Studie wurden mit bevölkerungsbasierten Referenzdaten der BELLA-Studie (*Be*fragung zum see*l*ischen Woh*l*befinden und Verh*a*lten [14]) vor der Pandemie verglichen. Nähere Informationen zum Design und zu den Methoden der COPSY-Studie sind publiziert [18, 19].

In diesem Beitrag werden die Ergebnisse der COPSY-Studie (2020–2022) zur Entwicklung der psychischen Gesundheit von Kindern und Jugendlichen während der Pandemie vorgestellt. Es wird über Resultate zu Risikogruppen für stärkere pandemiebedingte Belastungen und zu gesundheitlichen Ressourcen berichtet. Basierend auf den Daten zur psychischen Gesundheit, zu Risikofaktoren und Ressourcen werden evidenzbasierte Handlungsempfehlungen sowie Strategien für Präventions- und Interventionsansätze diskutiert.

## Pandemiebedingte Belastungen

Die Mehrheit der Kinder und Jugendlichen (70–80 %) gab zu allen 5 Erhebungszeitpunkten an, dass sie die Veränderungen im Zusammenhang mit der Pandemie als belastend empfinden. Besonders belastet fühlten sie sich im 2. Jahr der Pandemie (bis zu 83 %). Die meisten Kinder (76–83 %) fühlten sich durch die Einschränkung ihrer sozialen Kontakte belastet. Mehr als ein Drittel gaben Schwierigkeiten in der Beziehung zu Freund*innen an. Ungefähr ein Viertel berichtete, dass Streitigkeiten in der Familie zugenommen hatten. Etwa ein Drittel der Eltern sagte, dass familiäre Konflikte eher eskalierten als vor der Pandemie. Im Herbst 2022 nahmen die Belastungen mit den zunehmenden Lockerungen und sinkenden Infektionszahlen wieder ab (73 %).

### Lebensqualität und psychische Auffälligkeiten im Verlauf

Die gesundheitsbezogene Lebensqualität der Kinder und Jugendlichen verschlechterte sich v. a. im 1. Jahr der Pandemie deutlich. Vor der Pandemie betrug die Prävalenz für eine geminderte Lebensqualität 15 %. In den ersten beiden Befragungen war jeweils fast die Hälfte der Kinder und Jugendlichen betroffen (t1: 40 %; t2: 48 %). Nach fast 3 Jahren Pandemie (t5) waren es noch 27 % und damit nahezu doppelt so viele wie präpandemisch.

Die Prävalenz psychischer Auffälligkeiten stieg im 1. Jahr der Pandemie von präpandemisch 18 % auf 30 % (t1) bzw. 31 % (t2) an. Im 2. Jahr der Pandemie sank sie leicht ab und betrug nach fast 3 Jahren im Herbst 2022 (t5) noch 23 %. Der Verlauf bei den Symptomen für Ängstlichkeit und Depressivität war ähnlich. Insgesamt verbesserte sich die psychische Gesundheit von Kindern und Jugendlichen bis zum Herbst 2022 also wieder, psychische Auffälligkeiten waren aber immer noch häufiger als vor der Pandemie. Lediglich die Häufigkeit der Symptome für Depressivität war wieder auf das Niveau vor der Pandemie gesunken.

Die Prävalenz psychischer Auffälligkeiten betrug nach fast 3 Jahren Pandemie noch immer 23 %

**Figure 1:**
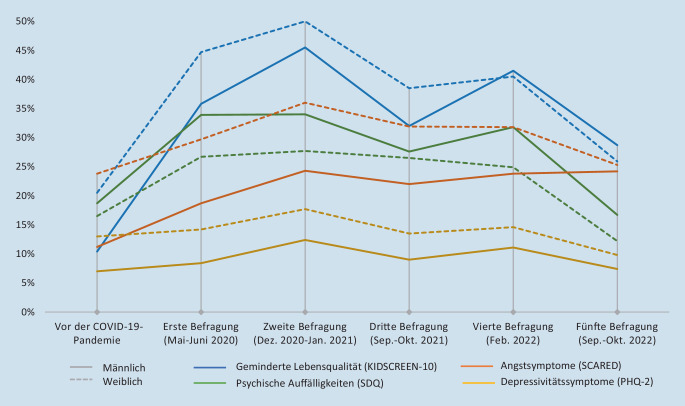

Mit Blick auf die untersuchten psychischen Gesundheitsvariablen waren Mädchen insgesamt häufiger belastet als Jungen (Abb. 1). Nur bei den psychischen Auffälligkeiten (gemessen mithilfe des Strengths and Difficulties Questionnaire [SDQ]) hatten Jungen mehr externalisierende Störungen als Mädchen.

Die Häufigkeit psychosomatischer Beschwerden wie Kopf- und Bauchschmerzen stieg im Verlauf der Pandemie kontinuierlich an. Reizbarkeit, Schlafprobleme, Niedergeschlagenheit und Nervosität nahmen im Herbst 2022 (t5) wieder etwas ab, waren aber immer noch deutlich stärker ausgeprägt als vor der Pandemie [10].

### Gesundheitsverhalten

Der Anteil der Kinder und Jugendlichen, die an weniger als 2 Tagen/Woche für mindestens 60 min am Tag körperlich aktiv sind (Indikator der WHO für zu geringe körperliche Aktivität [30]), stieg vom Sommer 2020 bis zum Winter 2020/2021 (t1 zu t2) von 35 auf 55 % an. In den folgenden 2 Jahren 2021 und 2022 nahm der Anteil wieder ab (t3: 30 %, t4: 36 %, t5: 27 %). Im 3. Pandemiejahr war der Anteil mit knapp einem Drittel immer noch 3‑mal höher als vor der Pandemie (Studie zur Gesundheit von Kindern und Jugendlichen in Deutschland [KIGGS] 2014–2017: 9 %, [8, 12, 24]). Analog nahm der Anteil derer, die an 3 bis 7 Tagen körperlich aktiv waren, im 1. Pandemiejahr um etwa ein Drittel ab (präpandemisch: 65 %, t1: 45 %). Im weiteren Pandemieverlauf stieg der Anteil wieder an (t3: 70 %, t4: 64 %, t5: 73 %).

### Risikofaktoren und Ressourcen

In der COPSY-Studie waren etwa 16 % der Kinder und Jugendlichen im Hinblick auf ihre psychische Gesundheit besonders vulnerabel. Risikofaktoren waren ein beengter Wohnraum und bezüglich der Eltern eine geringe Bildung, Migrationshintergrund, psychische Probleme oder hohe Belastungen durch die Pandemie. Kinder, die in eine solche Risikogruppe gehörten, hatten ein 2‑ bis 3-fach erhöhtes Risiko für eine geminderte Lebensqualität sowie ein 3‑ bis 5-fach erhöhtes Risiko für psychische Probleme. Besonders auffällig waren ein bis zu 2-fach erhöhtes Risiko für Ängste und ein 2‑ bis 4-fach erhöhtes Risiko für Depressivität.

Kinder mit Ressourcen hatten ein 5‑ bis 10-fach niedrigeres Risiko für eine geminderte Lebensqualität

In der COPSY-Studie wurden aber nicht nur Risikofaktoren in den Blick genommen, sondern auch schützende Ressourcen. Gute Ressourcen brachten rund 45 % der befragten Kinder und Jugendlichen mit und konnten sie den Risikofaktoren entgegensetzen. Solche Kinder verfügten über Problemlösefähigkeiten und Optimismus, fühlten sich von ihrem sozialen Umfeld gut unterstützt und erlebten die Familienatmosphäre positiv. Kinder mit diesen schützenden Faktoren hatten ein 5‑ bis 10-fach niedrigeres Risiko für eine geminderte Lebensqualität, psychische Probleme oder Depressivität. Das Risiko für Ängste war 3‑ bis 5-fach geringer im Vergleich zu denen, die diese Ressourcen nicht aufwiesen.

## Neue Krisen als Auslöser psychischer Belastungen

Die Ergebnisse zeigen, dass die Auswirkungen der Pandemie auf das Wohlbefinden und die psychische Gesundheit von Kindern und Jugendlichen nach 3 Jahren Pandemie etwas zurückgegangen sind. Gleichzeitig rücken neue Krisen in den Vordergrund. So gaben bei der 5. Befragung im Herbst 2022 noch 10 % der Heranwachsenden an, dass sie die Pandemie als besorgniserregend und seelisch belastend empfinden. Deutlich mehr Kinder und Jugendliche machten sich mittlerweile Sorgen um andere Krisen: Fast die Hälfte äußerte Ängste und Zukunftssorgen im Zusammenhang mit der Finanz- und Energiekrise sowie dem Ukraine-Krieg. Etwa ein Drittel gab seelische Belastungen hinsichtlich der Klimakrise an. Rund die Hälfte hatte Angst, dass die aktuellen Krisen noch lange anhalten, dass sich ihr Leben dadurch verschlechtert und sich ihre Familien bald weniger leisten können. Ein Drittel äußerte Ängste, dass sie durch die aktuellen Krisen zukünftig ihre Hobbys, ihren Schulabschluss oder ihren Wunschberuf nicht mehr ausführen könnten.

## Diskussion und Handlungsempfehlungen

Die COPSY-Studie zeigt auf, dass die Kinder und Jugendlichen in der COVID-19-Pandemie einerseits kurzfristig psychisch belastet waren, insbesondere im 1. Jahr und zu Zeiten des Lockdowns. Sie zeigt aber auch, dass knapp ein Drittel der Kinder und Jugendlichen nach 3 Jahren Pandemie immer noch unter psychischen Problemen und Beeinträchtigungen leidet. Besonders vulnerabel sind Kinder, die auf beengten Wohnraum leben, deren Eltern eine geringe Bildung, einen Migrationshintergrund, psychische Probleme oder hohe Belastungen durch die Pandemie aufwiesen [5, 13, 17].

Noch immer bewegt sich knapp ein Drittel der Kinder und Jugendlichen im Alltag zu wenig

Des Weiteren zeigt die COPSY-Studie, dass sich noch knapp ein Drittel der Kinder und Jugendlichen im Alltag zu wenig bewegen. Andere wichtige deutsche Studien wie etwa die KIDA-Studie weisen in eine ähnliche Richtung: Diese ergab, dass sich sogar 43 % der 3‑ bis 15-Jährigen nicht ausreichend bewegen [21]. Die Autoren der COPSY-Studie berichteten neben einem Bewegungsmangel auch von vermehrtem Medienkonsum und verschlechterten Ernährungsgewohnheiten in den ersten Pandemiejahren [18]. Andere Studien, etwa die groß angelegte DAK-Studie „Kinder- und Jugendgesundheit in Zeiten der Pandemie“, belegen die negativen Gesundheitseffekte: Die Zahlen für übergewichtige Kinder und Jugendliche stiegen während der Pandemie. Im Jahr 2021 waren unter den 15- bis 17-jährigen Jungen 15 % mehr adipös als 2019, bei den Mädchen waren es 6 % mehr. Bei den Mädchen wurde eine Zunahme an Essstörungen von 2019 bis 2021 um 54 % ermittelt [29].

Als eine der ersten Untersuchungen machen die Ergebnisse der COPSY-Studie deutlich, dass u. a. während der Schließungen von Kinderbetreuungs‑, Bildungs- und Freizeiteinrichtungen soziale Konflikte und Schwierigkeiten in der Familie und mit Freunden zunahmen – eine große Belastung für die Kinder und Jugendlichen. Als Folge der temporären Schließungen und der damit einhergehenden geringeren Förderung und Betreuung stehen wohl auch die Ergebnisse der Corona-KiTa-Studie. Diese stellte bei Kindern vor der Einschulung teilweise erheblich gestiegene Förderbedarfe in den Bereichen Sprache, Motorik und sozial-emotionale Entwicklung fest [11].

Vermehrte elterliche Belastungen beeinträchtigen wiederum das psychische Wohlbefinden der Kinder

Besonders Eltern jüngerer Kinder waren während der Pandemie stark belastet. Das zeigen bisherige COPSY-Publikationen sowie eine Studie des Bundesministeriums für Familien, Senioren, Frauen und Jugend [2, 18]. Die vermehrten Belastungen der Eltern hatten wiederum einen negativen Einfluss auf das psychische Wohlbefinden der Kinder [17]: Im 1. Pandemiejahr mit langen Lockdownzeiten stiegen nachweislich die Kindeswohlgefährdungen an [26] – vermutlich auch durch eine Überlastung mancher Eltern. Jugendliche tranken einer Online-Befragung des Bundesgesundheitsministeriums Anfang 2022 mit etwa 18.000 Teilnehmenden zwischen 14 und 21 Jahren zufolge zwar nicht mehr Alkohol, dafür stieg der Zigaretten- und Cannabiskonsum in dieser Altersgruppe an [9].

Nachdem die Bedrohungslage durch eine SARS-CoV-2-Infektion infolge von Impfungen und einer zunehmenden Herdenimmunität deutlich gesunken ist und Kinder und Jugendliche i. Allg. Infektionen relativ gut bewältigt haben, ist es an der Zeit, die psychischen, sozialen und körperlichen Langzeitfolgen der Pandemie für Kinder und Jugendliche in den Blick zu nehmen. Der Ethikrat und der Expert*innenrat der Bundesregierung [4, 6] betonen beide, dass die psychische Gesundheit der Kinder und Jugendlichen, die lange Zeit hinter dem Gesundheitsschutz der Älteren zurückstand, jetzt dringend gefördert werden müsse – insbesondere angesichts der bereits bestehenden neuen Krisen, die ein bis zwei Drittel der Jugendlichen schon jetzt belasten [19, 25]. Diese Forderung ist zentral im Hinblick darauf, dass psychische Auffälligkeiten in Kindheit und Jugend häufig bis ins Erwachsenenalter chronifizieren oder sich sogar zu manifesten psychischen Störungen entwickeln können. Werden psychische Auffälligkeiten nicht zeitnah behandelt, verlängert sich das individuelle Leiden erheblich. Zudem entstehen unnötige Kosten für das Gesundheitssystem.

Um die pandemiebedingten gesundheitlichen Folgen zu verringern und die Resilienz der Kinder und Jugendlichen zu stärken, können – auch im Hinblick auf aktuelle und zukünftige Krisen – aus der COPSY-Studie und der Literatur die in Tab. 1 aufgelisteten Gesundheitsempfehlungen zu Prävention und Interventionen abgeleitet werden. Ähnliche Empfehlungen finden sich auch im Abschlussbericht der interministeriellen Arbeitsgruppe „Gesundheitliche Auswirkungen auf Kinder und Jugendliche durch Corona“ [3].

**Table 1:** 

**Kleinkinder**
Sensibilisierung/Informationskampagnen für Eltern, Erzieher*innen und Pädiater*innen bezüglich möglicher frühkindlicher Entwicklungsverzögerungen im Bereich Sprache, Motorik sowie sozial-emotionale Fähigkeiten Aufbau eines bundesweiten psychischen Gesundheitsmonitorings zu Diagnostik und Einleitung der Behandlung *psychischer Auffälligkeiten,* z. B. durch sozialpädiatrische Zentren (SPZ) und Pädiater*innen („Frühe Hilfen“) in Vernetzung mit Erzieher*innen
**Schulkinder**
Aufbau eines bundesweiten psychischen Gesundheitsmonitorings zur Identifikation von *psychisch belasteten* Kindern und Jugendlichen in Schulen, Freizeit- und Sporteinrichtungen durch Lehrer*innen, Eltern, Schulpsycholog*innen, Freizeit‑/Sportvereinsmitarbeiter*innen Vermittlung von psychischen Gesundheitswissen an Schüler*innen, Lehrer*innen und Freizeit‑/Sportträger Zeitnaher Aufbau eines niedrigschwelligen, diskriminationsfreien Präventions‑/Interventionsangebots bei psychischen Problemen vor Ort (z. B. durch Ausbau des schulpsychologischen Dienstes und des BMFSFJ-Modellprogramms „Mental Health Coaches“ an Schulen) Personelle und finanzielle Stärkung der ambulanten Kinder- und Jugendpsychotherapeut*innen, Pädiater*innen und des SPZ sowie (teil-)stationärer Versorgung der Kinder- und Jugendmedizin/-psychiatrie/-psychosomatik Ausbau der Krisenintervention für Kinder und Jugendliche per Telefon (z. B. „Nummer gegen Kummer“: Tel.: 116111), online (bke-beratung.de) sowie wohnortnah und niedrigschwellig vor Ort (Kinder/Jugendkrisenberatungen)
**Allgemein**
Ausbau von kostenfreien, niedrigschwelligen und diskriminationsfreien *Bewegung*sangeboten für sozialschwache und übergewichtige/adipöse Kinder und Jugendliche, Förderung dieser durch Werbe‑/Gesundheitskampagnen (z. B. durch Krankenkassen) Aufklärungskampagnen zur *Medienkompetenz* und zum *Substanzkonsum* von Kindern, Jugendlichen und ihren Eltern (z. B. an Schulen) Ausbau von Anlaufstellen bei einer Suchttendenz (z. B. die Beratungsstelle „Lost in Space“ o. a. Suchtberatungsstellen) Ausbau des *Kinderschutzes* durch personelle und finanzielle Stärkung der Jugendämter, um Eskalationen, Gewalt und Übergriffe an Kindern und Jugendlichen in Familien insbesondere in Lockdown‑/Krisenzeiten schneller zu identifizieren und diese schützen zu können Zur Stärkung der Resilienz der Kinder und Jugendlichen: Ausweitung der Elternberatungen und Erziehungsberatungsstellen Zur Reduktion pandemiebedingter psychischer Belastungen der *Eltern: Unterstützungs*-/Gesundheitsangebote (z. B. Elternbegleiter vom BMFSFJ gefördert, Gesundheitskurse, Mutter‑/Vater-Kind-Kur etc.)

*BMFSFJ* Bundesministerium für Familie, Senioren, Frauen und Jugend

## Ausblick und aktuelle Forschungsaktivitäten

Als erste bundesweite bevölkerungsbasierte Längsschnittstudie legte die COPSY-Studie bereits früh zu Beginn der Pandemie den Fokus auf die psychische Gesundheit von Kindern und Jugendlichen. Die Ergebnisse haben ein starkes Echo in der Forschung und Versorgung ausgelöst. Es folgten viele weitere nationale Forschungsaktivitäten an verschiedenen Standorten in Deutschland. Dabei wurde deutlich, dass es bislang an einer bundesweiten Vernetzung von Studien zur Kindergesundheit fehlt. Zahlreiche Fachgesellschaften und Wissenschaftsgremien haben die bundesweite wissenschaftliche Erfassung der Pandemiefolgen und eines Gesundheitsmonitorings unter besonderer Berücksichtigung von Kindern und Jugendlichen empfohlen und gefordert.

Seit 2022 wird das interdisziplinäre NUM-Teilprojekt coverCHILD durch das BMBF gefördert

Im April 2020 wurde das Netzwerk Universitätsmedizin (NUM) als Teil des Krisenmanagements zur Bekämpfung der Pandemie gegründet. Im Fokus des NUM steht der Aufbau einer nachhaltigen nationalen Forschungsinfrastruktur, indem die Kompetenzen und Ressourcen von 36 Universitätskliniken in Deutschland gebündelt werden. Innerhalb des NUM entstand 2021 die Idee von Forscher*innen aus den Bereichen Pädiatrie, Kinder- und Jugendpsychiatrie und Child Public Health, sich diesem Netzwerk anzuschließen. Seit 2022 wird das interdisziplinäre NUM-Teilprojekt coverCHILD durch das Bundesministerium für Bildung und Forschung (BMBF) gefördert. Der Forschungsverbund coverCHILD hat zum Ziel, die Gesundheit von Kindern und Jugendlichen in gesundheitlichen und gesellschaftlichen Krisen wie etwa der COVID-19-Pandemie systematisch zu erforschen. Durch die Erhebung der COVID-19-bedingten psychischen und körperlichen Krankheitsfolgen, der kollateralen Auswirkungen und pandemiebedingten Einschränkungen für Kinder und Jugendliche generiert coverCHILD eine Evidenzbasis für die Prävention, Diagnostik und Behandlung von Krankheit und Gesundheit im Kindes- und Jugendalter. Nähere Informationen finden sich unter www.netzwerk-universitaetsmedizin.de und www.coverchild.de.

Anhand von Daten der COMO-Studie soll die Gesundheit von Kindern und Jugendlichen zielgerichtet gefördert werden

Darüber hinaus befasst sich das seit 2023 vom BMBF geförderte Verbundprojekt COMO mit den Auswirkungen der COVID-19-Pandemie auf die physische und psychische Gesundheit und das Gesundheitsverhalten von Kindern und Jugendlichen vor dem Hintergrund sozioökologischer Kontexte in Deutschland. Das Projekt COMO baut auf den bevölkerungsbasierten Studien COPSY und weiteren Surveys z. B. des Robert Koch-Instituts zu Motorik und Gesundheitsverhalten bei Kindern auf und vereint diese zu einer gemeinsamen interdisziplinären Längsschnittstudie. Anhand von neu erhobenen Daten zu den Veränderungen der körperlichen und seelischen Kindergesundheit sowie dem Gesundheitsverhalten in den Jahren 2023–2025 soll eine fundierte Datengrundlage für die Ableitung zielgerichteter Ansätze der Gesundheitsförderung, Prävention und Versorgung im Gesundheitswesen geschaffen werden.

Die Infrastrukturen und Erkenntnisse aus den Forschungsverbünden coverCHILD und COMO sollen auch in zukünftigen gesundheitlichen und gesellschaftlichen Krisen als Grundlage für ein vorausschauendes Krisen- und Pandemiemanagement genutzt werden können, um die Bedürfnisse und Interessen von Kindern und Jugendlichen zu schützen und eine frühzeitige Reaktion auf Herausforderungen zu ermöglichen.

## Fazit für die Praxis

- Die psychische Gesundheit von vielen Kindern und Jugendlichen hat sich während der Pandemie deutlich verschlechtert.
- Belastete Kinder und Jugendliche zu identifizieren, ist eine dringend notwendige gesamtgesellschaftliche Aufgabe. Dazu ist ein bundesweit einheitliches Monitoring der psychischen Folgen von Krisen für Kinder und Jugendlichen zu entwickeln und zu etablieren.
- Ein solches psychisches Gesundheits-Monitoring sollte von Erzieher*innen, Lehrer*innen, Schulpsycholog*innen, Kinderärzte*innen, Jugend- und Gesundheitsämtern flächendeckend durchführbar sein und genutzt werden können.
- Ziel sollte sein, so früh wie möglich Präventions- und Versorgungsbedarf festzustellen und zeitnahe Hilfen zur Verfügung zu stellen, sodass psychische Störungen vermieden werden.
- Das Monitoring ist auch notwendig, um zukünftiges Leid und damit einhergehende gesamtgesellschaftliche Krankheitskosten zu verringern.
- Eltern von besonders belasteten Kindern und Jugendlichen sollten zeitnah und unbürokratisch Unterstützung erhalten.
